# ChatGPT-3.5 Versus Google Bard: Which Large Language Model Responds Best to Commonly Asked Pregnancy Questions?

**DOI:** 10.7759/cureus.65543

**Published:** 2024-07-27

**Authors:** Keren Khromchenko, Sameeha Shaikh, Meghana Singh, Gregory Vurture, Rima A Rana, Jonathan D Baum

**Affiliations:** 1 Obstetrics and Gynecology, Hackensack Meridian Jersey Shore University Medical Center, Neptune, USA; 2 Obstetrics and Gynecology, Hackensack Meridian School of Medicine, Nutley, USA

**Keywords:** gemini, bard, commonly asked pregnancy questions, artificial intelligence, pregnancy, google bard, chatgpt-3.5

## Abstract

Large language models (LLM) have been widely used to provide information in many fields, including obstetrics and gynecology. Which model performs best in providing answers to commonly asked pregnancy questions is unknown. A qualitative analysis of Chat Generative Pre-Training Transformer Version 3.5 (ChatGPT-3.5) (OpenAI, Inc., San Francisco, California, United States) and Bard, recently renamed Google Gemini (Google LLC, Mountain View, California, United States), was performed in August of 2023. Each LLM was queried on 12 commonly asked pregnancy questions and asked for their references. Review and grading of the responses and references for both LLMs were performed by the co-authors individually and then as a group to formulate a consensus. Query responses were graded as “acceptable” or “not acceptable” based on correctness and completeness in comparison to American College of Obstetricians and Gynecologists (ACOG) publications, PubMed-indexed evidence, and clinical experience. References were classified as “verified,” “broken,” "irrelevant,” "non-existent,” and “no references.” Grades of “acceptable” were given to 58% of ChatGPT-3.5 responses (seven out of 12) and 83% of Bard responses (10 out of 12). In regard to references, ChatGPT-3.5 had reference issues in 100% of its references, and Bard had discrepancies in 8% of its references (one out of 12). When comparing ChatGPT-3.5 responses between May 2023 and August 2023, a change in ”acceptable” responses was noted: 50% versus 58%, respectively. Bard answered more questions correctly than ChatGPT-3.5 when queried on a small sample of commonly asked pregnancy questions. ChatGPT-3.5 performed poorly in terms of reference verification. The overall performance of ChatGPT-3.5 remained stable over time, with approximately one-half of responses being "acceptable" in both May and August of 2023. Both LLMs need further evaluation and vetting before being accepted as accurate and reliable sources of information for pregnant women.

## Introduction

Large language models (LLMs), a subset of artificial intelligence (AI), have shown tremendous growth and ability to provide human-like responses and information on a myriad of topics across industries, including the medical field. Chat Generative Pre-Training Transformer Version 3.5 (ChatGPT-3.5) (OpenAI, Inc., San Francisco, California, United States) was launched in November 2022, and its user base soared to 180.5 million users by August 2023 [1]. Bard, recently renamed Gemini (Google LLC, Mountain View, California, United States), reached an estimated one billion users with more than 140 million monthly visitors shortly after its launch in March 2023 [2]. Performance data on ChatGPT and Bard/Gemini in the medical field has been mixed. ChatGPT demonstrated clinical competence and general medical knowledge by passing the United States Medical Licensing Examination (USMLE) Steps One, Two, and Three, while Bard/Gemini displayed similar diagnostic capabilities as physicians in simulated patient scenarios [3-5]. However, ChatGPT performed poorly on open-ended and multiple-choice questions examined in the American Urological Association Self-Assessment Study Program, answering only 26.7% and 28.2% of questions correctly, respectively [6]. Additionally, both ChatGPT and Bard/Gemini achieved low scores in questions relating to endocrinology, diabetes, and diabetes technology, as both platforms obtained 58% correct responses [7].

Research on the role of LLMs in the field of obstetrics and gynecology (OBGYN) is limited. Grünebaum et al. concluded that ChatGPT has the potential to provide “preliminary information about virtually any topic” in OBGYN. However, they also acknowledge that the LLM has the potential to mislead and cause patient harm [8]. Wan et al. evaluated ChatGPT’s ability to answer commonly asked pregnancy questions and found this popular chatbot provided misinformation in 50% of queried questions and failed to provide proficient references 60% of the time [9]. These studies suggest that chatbots are not a safe alternative for consultation with a medical professional [8-10]. Despite this, studies have illustrated that pregnant women often turn to the Internet for information regarding nutrition, fetal development, and pregnancy complications. A recent survey of almost 400 pregnant women suggested that the Internet was almost as popular as asking a doctor for health information, registering 51% and 61%, respectively [11]. Another study of almost 200 women found that 84% of women used the Internet to retrieve information about pregnancy while they were pregnant [12]. In both of these studies, the authors also suggest that pregnant women often do not discuss online findings with their doctors, which is concerning [11,12].

Based on this evidence, it is likely that many pregnant patients are already utilizing LLMs such as ChatGPT and Bard/Gemini to gather medical information, but the validity of such information is unclear. The stockpile of evidence regarding AI chatbots continues to grow rapidly; however, heterogeneity in study design makes it difficult to draw any conclusions. Furthermore, the World Health Organization (WHO) issued an advisory statement that the “precipitous adoption of untested systems” could cause harm to patients and the future of AI [13]. Patients considering information or advice obtained from the Internet or any chatbot should consult their obstetric provider in order to safely guide clinical decision-making during pregnancy. 

The purpose of our study is to validate and compare the performance of ChatGPT-3.5 to that of Bard/Gemini as a source of information for commonly asked pregnancy questions and to verify the references they provide. 

Of note, this study was presented at American College of Obstetricians and Gynecologists (ACOG's) 2024 Annual Clinical and Scientific Meeting (2024 ACSM) on May 17th, 2024.

## Materials and methods

The study was conducted at Hackensack Meridian Jersey Shore University Medical Center in Neptune, United States. Our methods were adapted from Wan et al. with permission [9]. Wan et al. evaluated ChatGPT-3.5 responses to 12 commonly asked pregnancy questions in May 2023. Building upon their work, a similar qualitative analysis of ChatGPT-3.5 and Google Bard*, recently renamed Gemini, was performed using the same 12 commonly asked pregnancy questions. All queries for both LLMs were conducted and recorded in August 2023. Each LLM was accessed using a web-based interface. The basic format was “Can I (action) in pregnancy?” as we anticipate a pregnant woman might ask when seeking information regarding a specific activity as it relates to her pregnancy. Each queried question was followed up by the prompt “Tell me your references for this question” to record the LLM’s source for its response. The query questions are listed in Table 1.

**Table 1:** Common pregnancy questions used to query ChatGPT-3.5 and Google Bard.

Commonly asked pregnancy questions
Can I fly on a plane while pregnant?
Can I drink alcohol while pregnant?
Can I drink coffee while pregnant?
Can I eat deli meats while pregnant?
Can I dye my hair while pregnant?
Can I do laser hair removal while pregnant?
Is marijuana use safe during pregnancy?
Can I eat sushi while pregnant?
Can I vape while pregnant?
Can I do yoga while pregnant?
Can I go in a hot tub while pregnant?
What sleep position is best during pregnancy?

*Google Bard was renamed Gemini in February 2024. At the time that this study was conducted in August 2023, the LLM was known as Google Bard. For this reason, it will be referred to as Bard in the study moving forward.

Responses were recorded, reviewed, and graded by each co-author individually and then as a group to formulate a consensus. The co-authors are two board-certified OBGYN physicians (JDB, RAR), two OBGYN resident physicians (KK, GV), and two third-year medical students (SS, MS). Each member of the grading panel holds sufficient medical background to provide an honest review of each LLM's performance. American College of Obstetricians and Gynecologists (ACOG) Practice Bulletins and Committee Opinions were used as the default expert opinion (whenever available) against which ChatGPT-3.5 and Bard responses were compared. When ACOG guidance was not available, we used the best available evidence along with clinical judgment. 

Each response was evaluated and assigned a grade as defined in Table 2. A commentary on "not acceptable" responses is provided as appropriate. Examples of "acceptable" and "not acceptable" responses are demonstrated in the Appendices. Each reference provided was searched and validated using the Google search engine and then evaluated using the criteria listed in Table 2. Response performance and reference results of ChatGPT-3.5 and Bard were then compared. ChatGPT-3.5 responses and reference results from May 2023 and August 2023 were compared to evaluate its performance and degree of consistency over time. This study was exempt from Institutional Review Board (IRB) approval because no patient-level data were used. 

**Table 2:** LLM response grading scale and criteria (adapted from Wan et al. 2023 and Yeo 2023 with permission). LLM: large language models [9,14]

Grading scale	
Grade	Interpretation
Acceptable	Response is free from error and contains all necessary information.
Not acceptable	Response is missing necessary information (incomplete).
	Response contains factual errors (incorrect).
Criteria	
Reference issue	Explanation
Broken reference	Website address or an article DOI number was provided but the website’s page led to an error message and/or the DOI number was misleading. Digital Object Identifier (DOI) is a string of numbers, letters, or symbols used to uniquely identify an article or document on the Internet.
Irrelevant reference	List of references for a query that had little or nothing to do with the topic of interest.
Non-existent reference	References that do not exist.
No references	A list of references was not provided.
Verified reference	LLM provided verified reference(s).

## Results

Response performance

A grade of "acceptable" was given to 58% of ChatGPT-3.5 responses (seven out of 12) and 83% of Bard responses (10 out of 12). A grade of "not acceptable" was assigned to 42% of ChatGPT-3.5 responses with two "incomplete" and three "incorrect." Around 17% of Bard's responses were "not acceptable" due to being "incomplete." Appendices 1 and 2 have complete responses, consensus grades, and grade explanations for ChatGPT-3.5 and Bard, respectively. ChatGPT-3.5 and Google Bard Response Grading and Reference Evaluation are presented in Table 3 and Table 4, respectively.

**Table 3:** LLM response grading. ACOG: American College of Obstetricians and Gynecologists; AAP: American Academy of Pediatrics; LLM: large language models

Question	ChatGPT-3.5	Google Bard	Comment
Air travel	Not acceptable (incorrect)	Acceptable	ACOG states, “Most commercial airlines allow pregnant women to fly up to 36 weeks of gestation” [15]. ChatGPT-3.5 failed to mention this specific restriction and even mentioned that air travel up to 38 weeks was potentially allowed while Bard addressed it specifically and offered airline-specific recommendations.
Alcohol	Acceptable	Acceptable	ACOG, ChatGPT-3.5, and Bard strongly recommend against the use of alcohol and discuss fetal alcohol spectrum disorders (FASDs) [16,17]. Bard also offers some tips for avoiding alcohol use in pregnancy.
Caffeine	Acceptable	Acceptable	Both ChatGPT-3.5 and Bard are consistent with ACOG and provide a dose-limited recommendation, discuss adverse effects related to excess caffeine intake, and offer tips to reduce caffeine intake [18].
Deli meat	Acceptable	Acceptable	Both ChatGPT-3.5 and Bard are consistent with ACOG recommendations on Listeria and proper food safety guidelines [19]. Bard also provides more specific, safe alternatives to deli meats during pregnancy.
Hair dye	Acceptable	Acceptable	Both LLMs offer consistent recommendations with ACOG expert opinion on dyeing hair [20].
Laser hair removal	Not acceptable (incomplete)	Not acceptable (incomplete)	Both ChatGPT-3.5 and Bard omit evidence from a systematic review from 2019, which indicates that cutaneous laser treatment during pregnancy is safe for both the mother and fetus [21].
Marijuana use	Acceptable	Acceptable	ACOG, ChatGPT-3.5, and Bard all recommend against marijuana use while acknowledging potential risks to both the mother and fetus [22].
Sushi	Not acceptable (incorrect)	Acceptable	ChatGPT-3.5 incorrectly states that women can eat sushi during pregnancy. According to ACOG, it is recommended that pregnant individuals “avoid all raw and undercooked seafood, eggs, meat and poultry” and that they “do not eat sushi made with raw fish” [19,23]. Bard remains consistent with this recommendation.
Vaping	Acceptable	Acceptable	Both ChatGPT-3.5 and Bard advise against vaping while pregnant and discuss the risks of vaping during pregnancy. Bard provides tips for quitting vaping [24].
Yoga	Acceptable	Acceptable	Both LLMs correctly suggest that it is safe to practice yoga during pregnancy. They are cautious regarding certain poses but list methods and positions that may be beneficial. Polis et al. studied twenty-six yoga positions in healthy pregnant women in their third trimester. There were no adverse changes in maternal and fetal well-being during the session [25].
Hot tub	Not acceptable (incorrect)	Acceptable	Consistent with ACOG and AAP, both ChatGPT-3.5 and Bard caution against the use of hot tubs and saunas in pregnancy and the risk of increased body temperature during pregnancy [26-28]. However, ChatGPT-3.5 advised that hot tub time should be limited to under 15 minutes but did not provide a verified reference in regard to this time limit. Bard also provides options for ways for pregnant individuals to relax that are safe.
Sleep position	Not acceptable (incomplete)	Not acceptable (incomplete)	Both ChatGPT-3.5 and Bard do not include information from a prospective study on maternal sleeping position published in 2019 which concluded that “supine or non-left-sided sleep through 30 weeks gestation was not associated with adverse pregnancy outcomes linked to decreased placental blood flow in a large prospective cohort” [29].

**Table 4:** Reference evaluation.

Question	ChatGPT-3.5 references	Google Bard references
Air travel	No reference	Verified reference
Alcohol	Broken reference (2 of 4 provided)	Verified reference
Caffeine	No reference	Verified reference
Deli meat	No reference	Verified reference
Hair dye	No reference	Verified reference
Laser	No reference	Verified reference
Marijuana use	No reference	Broken reference (1 of 6 provided)
Sushi	No reference	Verified reference
Vaping	No reference	Verified reference
Yoga	Non-existent reference	Verified reference
Hot tub	No reference	Verified reference
Sleep position	No reference	Verified reference

ChatGPT-3.5 reference results

ChatGPT-3.5 was unable to provide verified references for most of the queried pregnancy-related questions. It provided no specific references for its responses on air travel, caffeine, deli meat, hair dye, laser hair removal, marijuana use, sushi, vaping, hot tub use, and sleep position. For the alcohol query, ChatGPT-3.5 provided two broken references, while the other two references were verified. In regard to yoga, ChatGPT-3.5 provided a non-existent reference. Overall, there were reference issues with 100% of the queries. When ChatGPT-3.5 could not provide a specific reference, it offered a variety of disclaimers listed in Appendix 3. 

Google Bard reference results

Bard offered verified references for responses regarding air travel, alcohol, caffeine, deli meat, hair dye, laser hair removal, sushi, vaping, yoga, hot tub, and sleep position. For the marijuana use query, Bard provided one broken reference and five verified references. Overall, Bard had reference deficiencies for 8% of its responses. 

ChatGPT-3.5 performance over time

When comparing ChatGPT-3.5 responses between the study conducted by Wan et al. in May 2023 and this present study conducted in August 2023, we observed a change in "acceptable" responses: 50% versus 58%, respectively (Table 5). Please refer to *ChatGPT: An Evaluation of AI-Generated Responses to Commonly Asked Pregnancy Questions *by Wan et al. 2023 for full ChatGPT-3.5 responses as queried in May 2023. Additionally, reference verification had worsened over time. In May 2023, 58% of the queries (seven out of 12) had reference deficiencies, and in August 2023, 100% of the queries demonstrated reference deficiencies. 

**Table 5:** ChatGPT-3.5 response comparison over time. ACOG: American College of Obstetricians and Gynecologists; AAP: American Academy of Pediatrics

Question	ChatGPT-3.5 - May 2023	ChatGPT-3.5 - August 2023	Comment
Air travel	Not acceptable (incomplete)	Not acceptable (incorrect)	Overall, August’s response improved from May's as it discussed specific gestational age restrictions whereas May did not provide any specific information; however, August was incorrect rather than incomplete as it included 38 weeks being the upper limit for possible air travel [15].
Alcohol	Acceptable	Acceptable	While the responses for both time points were different, they were both acceptable and consistent with ACOG [16,17].
Caffeine	Acceptable	Acceptable	While the responses for both time points were different, they were both acceptable and consistent with ACOG [18].
Deli meat	Acceptable	Acceptable	Both responses from May and August were similar to this query and were both acceptable and consistent with ACOG [19].
Hair dye	Acceptable	Acceptable	Both responses from May and August were similar to this query and were both acceptable and consistent with ACOG [20].
Laser hair removal	Not acceptable (incomplete)	Not acceptable (incomplete)	In May and August, ChatGPT-3.5 provided similar responses to this query but were found to be incomplete, as both time points omitted the systematic review from 2019 suggesting that laser hair removal is safe in pregnancy [21].
Marijuana use	Acceptable	Acceptable	While both time points provided acceptable responses, August was found to have a stronger response as it immediately mentioned that marijuana is not considered safe during pregnancy whereas May’s response was more ambivalent and mentioned that marijuana use in pregnancy was “a topic of ongoing debate and research” [22].
Sushi	Not acceptable (incorrect)	Not acceptable (incorrect)	May and August’s responses were different from each other and also not acceptable as they were incorrect. Both time points state that it is safe to eat sushi while pregnant, which is incorrect and inconsistent with ACOG [19,23].
Vaping	Not acceptable (incomplete)	Acceptable	In May and August, ChatGPT-3.5 provided different responses, with August’s response being acceptable and stronger than that of May. August explicitly mentions that it is strongly advised to avoid vaping during pregnancy while May provides a more ambivalent response [24].
Yoga	Not acceptable (incomplete)	Acceptable	Response by ChatGPT-3.5 to this question had improved over time. Compared to May’s response, ChatGPT-3.5's August response encourages focusing on gentle and restorative poses, listening to one’s body, staying hydrated, breathing and meditation, and wearing comfortable clothing. It also offers modifications and cautions against overheating [25].
Hot tub	Acceptable	Not acceptable (incorrect)	ChatGPT-3.5’s August response was found to be incorrect as it mentioned that pregnant women should limit their time in the hot tub to no more than 10-15 minutes, which is unverified, and thus, incorrect. May was different than that of August but acceptable and consistent with both ACOG and AAP guidelines [26-28].
Sleep position	Not acceptable (incomplete)	Not acceptable (incomplete)	In May and August, ChatGPT-3.5 provided different and incomplete responses. Both failed to include data from a 2019 prospective study regarding maternal sleeping position [29].

## Discussion

Overall, our study suggests that Bard was able to answer more questions correctly compared to ChatGPT-3.5 when responding to commonly asked pregnancy questions. ChatGPT-3.5’s deviation from evidence-based guidelines almost 50% of the time is a safety concern. Both chatbots were overly cautious in regard to laser hair removal and sleeping positions, which may discourage pregnant women from partaking in activities that have actually been shown to be safe [21,29].

We found that Bard’s ability to provide validated references was far superior to that of ChatGPT-3.5. For many of its responses, ChatGPT-3.5 offered no references but did provide a variety of disclaimers that patients should or may benefit from consultation with a medical professional. ChatGPT-3.5 is trained on a finite dataset up until September 2021, so perhaps it is unable to access all available references. ChatGPT-3.5’s striking omission of verifiable references calls into question its reliability. 

We compared ChatGPT-3.5 responses obtained in May 2023 by Wan et al. to responses obtained for this study in August 2023 [9]. The overall performance of ChatGPT-3.5 remained stable, with approximately one-half of responses being "acceptable"; however, more than two-thirds of responses changed between May and August. While the chatbot did improve for two questions (vaping and yoga), its performance worsened in regard to hot tub use. In fact, ChatGPT-3.5 offered potentially dangerous advice regarding the amount of time that a pregnant woman could safely spend in a hot tub. This lack of consistency is of particular concern as the typical end-user is most likely looking for guidance and not seeking information that varies widely or needs verification.

This study adds to the literature by maintaining a consistent grading system and including a diverse, medical background review panel. We reviewed the responses using a panel of both medical professionals and students. In addition, we offered a unique perspective on the LLMs’ reproducibility over time by assessing ChatGPT-3.5 response performance over a few months. The study acknowledged some change in response accuracy and a worsening in reference verification. The responses over time deferred without any obvious changes in evidence or recommendations since September 2021. This is concerning when it comes to ascertaining medical information.

Several limitations must be considered. We chose to use ChatGPT-3.5, as opposed to a newer version, because it was freely available to the public. We acknowledge the more up-to-date version, ChatGPT-4.0, has shown notable performance improvement and outperformed ChatGPT-3.5 on both the Scholastic Assessment Test (SAT) and Bar Exam [30]. LLM chatbots are rapidly evolving, but vetting of the models is lagging. It is reasonable to assume that improved versions are, in fact, improved. However, each version must be tested, especially when it comes to the dissemination of medical information. In addition, our qualitative analysis is appropriate but makes performing statistical analysis difficult due to our shorter list of pregnancy-related questions. For the purpose of this study, we believe a small sampling of questions provides a manageable introduction to the rapidly evolving technology and LLM use in the field of OBGYN. We recognize that larger studies would facilitate an even better understanding of LLM performance.

Another element that may influence response output is the verbage used to query the chatbots. In other words, the LLMs have the potential to change their answers depending on how the question is phrased. Variations in LLM performance when compared against one another or when queried by different users at different times could be the result of being trained on different datasets, internet access, and user interactions. These factors cannot always be accounted for or controlled for, which is why checking an LLM's ability to answer commonly asked pregnancy questions (or any set of questions) is so important prior to acceptance of these chatbots for use for this purpose. Future studies using newer versions of ChatGPT and Gemini or evaluating the effects of different inputs for pregnancy-related questions may lead to different conclusions regarding LLM performance. 

Our methodology, transparency, and diverse review panel allow for this study to be reproducible. We have made verbatim responses of both LLMs available to all readers. They can be used as examples for those who choose to replicate our study and grade their own response outputs. In addition, we included the verbatim responses to allow readers to judge the responses themselves. We invite future investigation and commentary in response to our study.

## Conclusions

LLMs have quickly become a worldwide phenomenon, available to anyone with internet access. Their ability to perform on medical board examinations and standardized aptitude tests demonstrates their remarkable potential. Given the popularity of LLMs, it is reasonable to assume that pregnant women are turning to AI chatbots to search for information regarding their pregnancy. Our study adds to an emerging body of literature regarding the role of chatbots in pregnancy. We believe this to be the first report of its kind to compare the most commonly used AI chatbots, ChatGPT-3.5 and Bard/Gemini.

We observed that Bard answered more questions correctly than ChatGPT-3.5 when queried on commonly asked pregnancy questions. ChatGPT-3.5 performed poorly in regard to reference verification. Observed over time, ChatGPT-3.5's performance changed but overall remained disappointing and its reference verification worsened. Both LLMs need further evaluation and vetting before being accepted as accurate and reliable for providing pregnancy-related information.

## Appendices

Appendix 1: questions, ChatGPT-3.5 response, grade, and explanation

1. Can I Fly on a Plane While Pregnant?

Yes, in most cases, you can fly on a plane while pregnant. However, there are some considerations and precautions you should keep in mind: 

Consult your healthcare provider: Before making any travel plans, it's important to consult your healthcare provider. They can assess your individual pregnancy and advise you on whether it's safe for you to fly, taking into account factors such as how far along you are, any complications, and your overall health. 

Gestational age: Generally, flying during the first and second trimesters is considered safer and more comfortable than flying during the third trimester. Many airlines have restrictions on flying after a certain gestational age (often around 36-38 weeks), so be sure to check with the airline before booking your flight. 

Medical records: Some airlines might require a letter from your healthcare provider stating that you're fit to travel, especially if you're in the later stages of pregnancy. It's a good idea to have your medical records and any necessary documentation with you. 

Comfort and safety: Long flights can be uncomfortable for anyone, and pregnancy can add to this discomfort. Make sure you have enough space to move around and stretch your legs. Additionally, consider the risk of deep vein thrombosis (DVT), a condition where blood clots can form in your legs during long periods of inactivity. To reduce this risk, stay hydrated, move around the cabin periodically, and do ankle exercises. 

Radiation exposure: The radiation exposure during a single flight is generally considered safe for pregnant women. However, if you're a frequent flyer or plan to take multiple flights, discuss this with your healthcare provider. 

Air pressure and oxygen levels: Commercial aircraft cabins are pressurized to make them comfortable for passengers, but the cabin air pressure is lower than what you experience at ground level. This reduction in pressure can affect oxygen levels. While this is generally not an issue for most pregnant women, those with certain medical conditions should discuss this with their healthcare provider. 

Hydration and nutrition: Airplane cabins can be dry, so it's important to stay hydrated. Pack nutritious snacks to keep your energy levels up during the flight. 

Precautions for high-risk pregnancies: If you have a high-risk pregnancy due to factors like preeclampsia, placental problems, or a history of preterm labor, your healthcare provider might advise against flying. 

Remember, every pregnancy is unique, and the decision to fly should be based on your individual circumstances and the advice of your healthcare provider. Always prioritize your health and the health of your baby when making travel decisions during pregnancy.

Grade: Not acceptable (incorrect)

We found ChatGPT-3.5’s air travel response to be “not acceptable” because it is "incorrect." Its use of the statement that “many airlines have restrictions on flying after a certain gestational age (often around 36-38 weeks)” is "incorrect,"  as ACOG guidelines explicitly state that “most commercial airlines allow pregnant women to fly up to 36 weeks of gestation” [15]. ChatGPT-3.5’s inaccurate recommendation could result in potentially unsafe air travel after 36 weeks of pregnancy. ChatGPT-3.5’s response was “not acceptable” in both May and August queries. In fact, we believe ChatGPT-3.5’s most recent response represents misinformation.

2. Can I Drink Alcohol While Pregnant?

I am not a doctor, but I can provide some general information on this topic. It is generally advised that pregnant individuals avoid alcohol consumption during pregnancy. There is no known safe level of alcohol consumption during pregnancy, as alcohol can pass from the mother's bloodstream to the developing fetus through the placenta. 

Drinking alcohol during pregnancy can increase the risk of various complications, including fetal alcohol spectrum disorders (FASDs), which can lead to physical, behavioral, and cognitive issues in the child. These effects can be long-lasting and have a significant impact on the child's development. 

If you are pregnant or planning to become pregnant, it's important to consult with a qualified healthcare professional, such as an obstetrician or a prenatal care provider. They can provide personalized guidance and recommendations based on your specific situation and medical history. 

Remember, the well-being of both you and your baby is a top priority, so it's best to err on the side of caution when it comes to alcohol consumption during pregnancy.

Grade: Acceptable

3. Can I Drink Coffee While Pregnant?

While it's generally safe to consume moderate amounts of caffeine during pregnancy, it's important to be cautious and mindful of your caffeine intake. The American College of Obstetricians and Gynecologists and other health organizations recommend that pregnant individuals limit their caffeine intake to about 200 milligrams (mg) per day, which is roughly equivalent to a 12-ounce cup of coffee. 

Excessive caffeine intake during pregnancy has been associated with an increased risk of miscarriage and preterm birth. It's also important to note that caffeine is a stimulant that can cross the placenta and affect the developing fetus. High caffeine intake can lead to fetal growth restriction and potentially increase the risk of developmental issues. 

If you're a coffee lover and you're pregnant, here are a few things to keep in mind: 

Limit your intake: Aim to consume no more than 200 mg of caffeine per day. Remember that caffeine is found in other sources besides coffee, such as tea, chocolate, and some soft drinks. 

Choose your coffee wisely: Different types of coffee have varying levels of caffeine. For instance, espresso generally contains more caffeine per ounce than regular brewed coffee. Be mindful of the type and size of coffee you're drinking.

Decaffeinated options: If you're concerned about your caffeine intake, you can opt for decaffeinated coffee, which has had most of the caffeine removed. 

Stay hydrated: Remember to drink plenty of water throughout the day to stay hydrated. 

Consult your healthcare provider: It's always a good idea to discuss your caffeine consumption with your healthcare provider. They can provide personalized advice based on your health status and pregnancy. 

Listen to your body: If you notice any adverse reactions or discomfort after consuming caffeine, consider reducing your intake or eliminating it altogether. 

Remember that every pregnancy is different, and individual sensitivities to caffeine can vary. It's best to err on the side of caution and follow the recommendations of your healthcare provider. 

Grade: Acceptable

4. Can I Eat Deli Meats While Pregnant?

Yes, you can eat deli meats while pregnant, but there are some precautions you should be aware of to ensure your safety and the safety of your baby. Deli meats, such as ham, turkey, chicken, and salami, can sometimes carry a bacteria called Listeria monocytogenes, which can lead to a foodborne illness called listeriosis. Listeriosis can be harmful to both you and your baby, as it may increase the risk of miscarriage, stillbirth, preterm labor, or serious health problems for the newborn. 

To reduce the risk of listeriosis, here are some guidelines you can follow when consuming deli meats during pregnancy: 

Heat: It's generally recommended to heat deli meats until they are steaming hot before eating them. Listeria bacteria are killed by heat, so by heating the deli meats, you can minimize the risk of infection. 

Avoid cold and raw: Avoid consuming cold deli meats or those that are served raw, as these have a higher risk of containing Listeria. This includes sandwiches with deli meats, cold salads with meat, and unpasteurized cured meats. 

Check labels: If you choose to consume deli meats, make sure they are from a reputable source and have been properly processed and handled. Look for labels indicating that the product is pasteurized or heated. 

Freshness: Consume deli meats promptly and check for any signs of spoilage, such as an off smell or unusual appearance. 

Opt for alternatives: If you're concerned about the risks associated with deli meats, you can consider alternatives like cooked meats, poultry, or plant-based protein sources. 

Remember to consult with your healthcare provider before making any dietary changes during pregnancy. They can provide personalized advice based on your health and medical history.

Grade: Acceptable

5. Can I Dye My Hair While Pregnant?

I am not a medical professional, but I can offer you some general information on this topic. It's always recommended to consult with your healthcare provider before making any decisions during pregnancy. 

Hair dye and other chemical treatments contain various ingredients that are absorbed through the skin. While many hair dyes are considered safe to use during pregnancy, some studies suggest that only limited amounts of these chemicals are actually absorbed and that the risk of harm to the fetus is low. However, the research in this area is not extensive, and opinions can vary among healthcare professionals. 

If you're considering dyeing your hair while pregnant, here are a few things to keep in mind: 

Consult your healthcare provider: Before using any hair dye or chemical treatment, it's best to consult your obstetrician, midwife, or healthcare provider. They can provide personalized advice based on your individual health, stage of pregnancy, and any preexisting conditions. 

Choose safer options: Consider using hair dyes that are ammonia-free and have fewer potentially harmful chemicals. These products are generally considered to be less harsh on the scalp and may pose a lower risk. 

Ventilation: Ensure that the area where you're applying the hair dye is well-ventilated to reduce the inhalation of fumes. 

Patch test: Perform a patch test before applying the hair dye to your entire scalp. This can help you identify any allergic reactions or adverse effects before using the product more extensively. 

Limit skin contact: If you decide to dye your hair, try to minimize the dye's contact with your scalp and skin. Using gloves and avoiding excessive absorption through the scalp may reduce potential risks. 

Temporary and semi-permanent dyes: Temporary and semi-permanent dyes are generally considered safer options because they sit on the surface of the hair and have fewer chemicals that can be absorbed into the body. 

Remember that pregnancy is a sensitive time, and even if the risks are considered low, it's always wise to err on the side of caution. Your healthcare provider can provide the most relevant guidance for your situation, taking into account any potential risk factors or concerns you may have.

Grade: Acceptable

6. Can I Do Laser Hair Removal While Pregnant?

I am not a medical professional, but I can provide some general information on this topic. It's important to consult with a qualified healthcare provider before making any decisions about undergoing laser hair removal while pregnant. 

Laser hair removal involves the use of intense light to target hair follicles and inhibit hair growth. While there is limited research on the safety of laser hair removal during pregnancy, most healthcare professionals advise against undergoing the procedure while pregnant. This is primarily due to concerns about potential risks to the developing fetus. 

During pregnancy, hormonal changes can affect your skin's sensitivity and response to treatments. Additionally, there's a possibility that the energy from the laser could be absorbed by the skin in a way that might affect the fetus. Although the risk is considered low, it's generally recommended to avoid unnecessary procedures during pregnancy, especially those that involve exposure to intense light or heat. 

If you're interested in laser hair removal, it's best to wait until after pregnancy and consult with a dermatologist or healthcare provider for personalized advice based on your specific situation. They can help you weigh the potential risks and benefits and guide you in making the safest decision for you and your baby. 

Grade: Not acceptable (incomplete)

We found ChatGPT-3.5’s response to the laser hair removal query to be “not acceptable” because it is “incomplete.” It omits a systematic review from 2019 on the safety of cutaneous laser treatment during pregnancy and thus deprives women of necessary information upon which they can make more informed decisions regarding laser hair therapy during pregnancy [9,21].

7. Is Marijuana Use Safe in Pregnancy?

No, marijuana is not considered safe during pregnancy. The active compound in marijuana, delta-9-tetrahydrocannabinol (THC), can cross the placenta and affect the developing fetus. Research suggests that using marijuana during pregnancy can pose various risks to both the mother and the baby. 

Fetal development: Marijuana use during pregnancy has been associated with negative effects on fetal brain development, potentially leading to cognitive and behavioral problems later in the child's life. 

Low birth weight: Babies born to mothers who used marijuana during pregnancy are more likely to have a lower birth weight, which can lead to various health issues. 

Preterm birth: There's evidence to suggest that marijuana use during pregnancy may increase the risk of preterm birth, which can lead to a range of complications for the baby. 

Cognitive and behavioral issues: Children exposed to marijuana in utero may be at a higher risk of cognitive and behavioral problems, including issues with attention, memory, and learning. 

Respiratory issues: Smoking marijuana can expose both the mother and the baby to harmful chemicals, which can potentially lead to respiratory problems. 

Long-term effects: While more research is needed, some studies have indicated that prenatal marijuana exposure could have long-term effects on the child's development and behavior. 

Given these potential risks, many health organizations, including the American College of Obstetricians and Gynecologists and the American Academy of Pediatrics, recommend that pregnant individuals avoid using marijuana. If you're pregnant and have questions about substances to avoid or any aspect of your health, it's important to consult with a healthcare professional for guidance tailored to your situation.

Grade: Acceptable

8. Can I Eat Sushi While Pregnant?

Yes, you can eat sushi while pregnant, but there are some precautions you should take to ensure your safety and the health of your baby. The main concern with eating sushi during pregnancy is the risk of consuming raw or undercooked fish, which could potentially contain harmful bacteria or parasites. 

Here are some guidelines to follow: 

Choose safe options: Opt for sushi that uses cooked seafood, such as shrimp, crab, or fully cooked eel. Also, vegetarian options like avocado, cucumber, and sweet potato are safe choices. 

Avoid high mercury fish: Certain types of fish are high in mercury, which can be harmful to your developing baby's nervous system. Avoid fish like shark, swordfish, king mackerel, and tilefish. 

Limit raw fish: If you want to eat raw fish, make sure it's from a reputable source with strict quality control measures. Some pregnant women prefer to avoid raw fish altogether due to the potential risks. 

Check for freshness: Sushi should be made with fresh ingredients. If you're unsure about the quality of a sushi restaurant, it might be better to avoid it during pregnancy. 

Maintain proper hygiene: Wash your hands before eating and ensure that the utensils, plates, and surfaces are clean. 

Moderation: As with any type of food, moderation is key. Don't overindulge in sushi during pregnancy. 

Consult your healthcare provider: It's always a good idea to consult your healthcare provider before making dietary changes during pregnancy. They can provide personalized advice based on your medical history and individual circumstances. 

Remember that the guidelines provided here are general recommendations, and every pregnancy is unique. It's important to prioritize your health and the health of your baby. If you have concerns or questions, it's best to consult with your healthcare provider for specific guidance tailored to your situation.

Grade: Not acceptable (incorrect)

We found ChatGPT-3.5’s response to the query regarding sushi consumption in pregnancy to be “incorrect.” It states that pregnant individuals can eat sushi, which contradicts ACOG’s recommendation to avoid all raw and undercooked seafood, eggs, meat, and poultry in pregnancy [9,19,23].

9. Can I Vape While Pregnant?

It is strongly advised to avoid vaping or any form of nicotine or tobacco use during pregnancy. Nicotine and the other harmful chemicals present in vaping products can have negative effects on both the pregnant person and the developing fetus. 

Nicotine is known to constrict blood vessels, which can reduce the flow of oxygen and nutrients to the fetus, potentially leading to developmental problems. It can also contribute to preterm birth, low birth weight, and other complications. In addition, the various chemicals and additives found in vaping products may pose risks to the baby's health and development. 

If you are pregnant and struggling with nicotine addiction, it's important to seek guidance from a healthcare professional. They can provide you with support and resources to quit smoking or vaping safely. Remember that the health and well-being of both you and your baby are the top priorities during pregnancy, and avoiding harmful substances is crucial.

Grade: Acceptable

10. Can I Do Yoga While Pregnant?

Yes, in most cases, practicing yoga during pregnancy can be beneficial for both the mother and the baby. However, there are certain precautions and guidelines you should follow to ensure safety and to make the practice appropriate for your changing body. 

Here are some tips for practicing yoga during pregnancy: 

Consult your healthcare provider: Before starting any new exercise routine, including yoga, it's important to consult your healthcare provider or obstetrician. They can give you personalized advice based on your health status and any potential complications. 

Choose prenatal yoga classes: Look for prenatal yoga classes specifically designed for pregnant women. These classes are led by instructors who are trained to modify poses and sequences to accommodate the needs of pregnant participants. 

Avoid certain poses: Some yoga poses are not suitable for pregnant women due to the potential strain they might put on the abdominal area or the risk of falling. Poses that involve deep twists, backbends, and inversions should be approached with caution or avoided. 

Focus on gentle and restorative poses: Prenatal yoga typically emphasizes gentle and restorative poses that help with flexibility, relaxation, and improving circulation. These poses can help relieve common discomforts associated with pregnancy. 

Listen to your body: Your body is going through significant changes during pregnancy. Listen to your body and practice within your comfort zone. Avoid pushing yourself too hard or trying to achieve advanced poses. 

Modify as needed: As your body changes, you might need to modify certain poses to accommodate your growing belly and changing balance. Use props such as blocks, bolsters, and straps to support your practice. 

Stay hydrated: Pregnancy can lead to increased dehydration, so be sure to drink plenty of water before, during, and after your yoga practice. 

Breathing and meditation: Incorporate breathing exercises (pranayama) and meditation into your practice. These techniques can help reduce stress, improve relaxation, and provide emotional support during pregnancy. 

Avoid overheating: Pregnancy can make you more sensitive to heat. Practice in a well-ventilated space and avoid hot yoga classes or practicing in excessively hot conditions. 

Wear comfortable clothing: Wear comfortable, breathable clothing that allows for easy movement. A good supportive maternity yoga outfit can help you feel at ease during your practice. 

Remember, every pregnancy is unique, and what works for one person might not work for another. It's important to prioritize your safety and well-being. If you experience any discomfort, pain, or unusual symptoms during your yoga practice, stop immediately and consult your healthcare provider. 

In summary, practicing yoga during pregnancy can be a wonderful way to stay active, relieve stress, and promote overall well-being. Just be sure to follow the guidance of your healthcare provider and choose appropriate prenatal yoga classes or modifications. 

Grade: Acceptable

11. Can I Go in a Hot Tub While Pregnant?

I am not a medical professional, but I can provide some general information. It's important to consult with your healthcare provider before making any decisions about activities during pregnancy, including using a hot tub. 

Hot tubs, saunas, and hot baths can raise your body temperature, and during pregnancy, maintaining a stable body temperature is crucial for the health of both you and your baby. Elevated body temperature, especially in the early stages of pregnancy, has been associated with an increased risk of birth defects and other complications. 

Pregnant women are generally advised to avoid activities that could raise their core body temperature significantly, such as sitting in hot tubs or saunas for extended periods of time. It's recommended that the water temperature in a hot tub be below 100°F (37.8°C) if pregnant women choose to use it, and they should limit their time in the hot tub to no more than 10-15 minutes. 

However, every pregnancy is unique, and what might be safe for one person might not be safe for another due to individual health considerations. If you're considering using a hot tub while pregnant, it's absolutely essential to consult your healthcare provider first. They can provide personalized advice based on your medical history, the stage of your pregnancy, and any other relevant factors. 

Remember that your health and the health of your baby are top priorities, so always err on the side of caution and follow the guidance of your healthcare provider.

Grade: Not acceptable (incorrect)

We found ChatGPT-3.5’s hot tub response to be “not acceptable” because it is “incorrect.” ChatGPT-3.5 is not able to provide a verified reference for its statement that pregnant women should limit their time in the hot tub to no more than 10-15 minutes [26-28].

12. What Sleep Position Is Best During Pregnancy?

During pregnancy, the best sleep position changes as the pregnancy progresses. Here's a general guideline for each trimester: 

First trimester: During the first trimester, sleeping on your back or side is generally considered safe. However, sleeping on your left side is often recommended. This position improves blood circulation to the heart and allows for better blood flow to the fetus and uterus. It can also help alleviate pressure on the back and reduce the risk of backaches and hemorrhoids. 

Second trimester: As your pregnancy advances, it's still recommended to sleep on your side, preferably the left side. Sleeping on your left side continues to facilitate good blood circulation and reduces the risk of compressing the inferior vena cava, a large vein that returns blood from the lower body to the heart. 

Third trimester: Sleeping on your left side remains the preferred position. This position can help prevent the uterus from putting excessive pressure on the liver, which is located on the right side of the body. It can also improve kidney function and decrease the likelihood of swelling in the legs and ankles. 

It's generally recommended to avoid sleeping on your back during the later stages of pregnancy, particularly after the first trimester. Sleeping on your back can lead to the weight of the uterus pressing on the vena cava, potentially causing dizziness, low blood pressure, and reduced blood flow to both you and your baby. 

If you find it uncomfortable to sleep on your left side for extended periods, you can also try sleeping on your right side. Using pillows to support your body and finding a comfortable arrangement can help you maintain a suitable sleep position throughout your pregnancy. 

Always consult your healthcare provider for personalized advice based on your specific circumstances, as individual factors can influence the best sleep position for you during pregnancy.

Grade: Not acceptable (incomplete)

We found ChatGPT-3.5’s response regarding sleep position to be “not acceptable” as it is “incomplete.” The response omits a prospective evaluation of maternal sleeping position from 2019 and has the potential to mislead pregnant individuals as the study found that supine and non-left lateral sleep through 30 weeks was not associated with adverse pregnancy outcomes [9,29].

Appendix 2: questions, Google Bard response, grade, and explanation

1. Can I Fly on a Plane While Pregnant?

Yes, you can fly on a plane while pregnant, as long as your pregnancy is healthy and uncomplicated. Most airlines allow pregnant women to fly domestically until about 36 weeks of pregnancy. For international flights, the cut-off may be earlier. Check with your airline before you book your flight. 

Here are some things to keep in mind if you are planning to fly while pregnant: 

Talk to your doctor before you fly. They can advise you on whether it is safe for you to fly and give you any specific recommendations. 

Make sure you have plenty of fluids to drink during the flight. Dehydration can increase the risk of complications. 

Get up and move around every few hours to avoid blood clots. 

Wear comfortable clothing and shoes. 

Bring a pillow and blanket to help you get comfortable. 

Let the flight attendant know that you are pregnant so they can make sure you have any special needs. 

Here are some airlines that allow pregnant women to fly: 

Air Canada: 36 weeks of pregnancy for domestic flights, 32 weeks of pregnancy for international flights 

Alaska Airlines: 36 weeks of pregnancy for domestic flights, 32 weeks of pregnancy for international flights 

American Airlines: 36 weeks of pregnancy for domestic flights, 32 weeks of pregnancy for international flights 

Delta Airlines: Throughout entire pregnancy 

Emirates: 35 weeks of pregnancy for single pregnancies, 31 weeks of pregnancy for multiple pregnancies 

Frontier Airlines: 36 weeks of pregnancy for domestic flights, 32 weeks of pregnancy for international flights 

Hawaiian Airlines: 36 weeks of pregnancy for domestic flights, 32 weeks of pregnancy for international flights 

JetBlue Airways: 36 weeks of pregnancy for domestic flights, 32 weeks of pregnancy for international flights 

Lufthansa: Throughout entire pregnancy 

Southwest Airlines: 36 weeks of pregnancy for domestic flights, 32 weeks of pregnancy for international flights 

United Airlines: 36 weeks of pregnancy for domestic flights, 32 weeks of pregnancy for international flights 

If you have any questions or concerns about flying while pregnant, be sure to talk to your doctor.

Grade: Acceptable

2. Can I Drink Alcohol While Pregnant?

No, it is not safe to drink alcohol at any time during pregnancy. There is no known safe amount of alcohol use during pregnancy or while trying to get pregnant. All types of alcohol are equally harmful, including all wines and beer. 

Alcohol can cross the placenta and reach the baby's bloodstream. It can interfere with the development of the baby's brain and other critical organs, structures, and physiological systems. Prenatal alcohol exposure is a leading preventable cause of birth defects and neurodevelopmental abnormalities in the United States. 

The effects of alcohol on a developing baby can range from mild to severe, depending on the amount of alcohol the mother drinks and how often she drinks. Even small amounts of alcohol can increase the risk of miscarriage, premature birth, and low birth weight. Drinking heavily during pregnancy can cause a serious condition called fetal alcohol spectrum disorder (FASD). FASD can cause a wide range of problems, including physical and intellectual disabilities, learning and behavioral problems, and problems with attention and impulse control. 

If you are pregnant or planning to become pregnant, it is important to avoid alcohol completely. There is no safe amount of alcohol use during pregnancy. If you are struggling to quit drinking, talk to your doctor or a healthcare professional. There are many resources available to help you quit drinking and stay sober during pregnancy. 

Here are some tips for avoiding alcohol during pregnancy: 

Don't drink any alcohol, even if you think it's a small amount. 

Avoid situations where you might be tempted to drink, such as parties or social gatherings. 

Talk to your friends and family about your decision to avoid alcohol and ask for their support. 

Drink plenty of water and other non-alcoholic beverages. 

There are many non-alcoholic alternatives to alcoholic drinks available, such as mocktails and NA beer. 

If you are concerned about your alcohol use during pregnancy, talk to your doctor. They can help you assess your risk and develop a plan to quit drinking safely.

Grade: Acceptable

3. Can I Drink Coffee While Pregnant?

Yes, you can still enjoy a mug of coffee every now and then during your pregnancy. Just make sure that you don't have more than 200mg of caffeine in a day. This is about the amount in 1½ 8-ounce cups of coffee or one 12-ounce cup of coffee. 

Caffeine is a stimulant that can cross the placenta and reach the baby. It can affect the baby's heart rate and sleep patterns. Too much caffeine during pregnancy has been linked to a number of problems, including: 

Miscarriage 

Low birth weight 

Premature birth 

Stillbirth 

Increased risk of Sudden Infant Death Syndrome (SIDS) 

If you are pregnant, it is best to limit your caffeine intake as much as possible. There are many other ways to get the energy you need without caffeine, such as eating healthy foods, getting enough sleep, and exercising regularly. Here are some tips for reducing your caffeine intake: 

Choose decaffeinated coffee or tea. 

Drink water instead of sugary drinks that contain caffeine, such as soda and energy drinks. 

Limit your intake of chocolate, which also contains caffeine. 

Be aware of the caffeine content of other foods and beverages, such as energy bars and colas. 

If you are concerned about your caffeine intake, talk to your doctor. They can help you create a plan that is safe for you and your baby. 

Grade: Acceptable

4. Can I Eat Deli Meats While Pregnant?

It is best to avoid deli meats while pregnant, as they can harbor Listeria bacteria, which can cause food poisoning. Listeria is especially dangerous for pregnant women because it can cross the placenta and infect the fetus, which can lead to miscarriage, stillbirth, or serious health problems. 

The Centers for Disease Control and Prevention (CDC) recommends that pregnant women avoid deli meats altogether. If you do choose to eat deli meats, be sure to heat them to an internal temperature of 165 degrees Fahrenheit (74 degrees Celsius) to kill any harmful bacteria. You should also avoid eating deli meats that have been left out at room temperature for more than two hours. 

Here are some safe alternatives to deli meats during pregnancy: 

Cooked chicken, turkey, or ham 

Tuna or salmon that has been canned or cooked 

Hard-boiled eggs 

Cheese 

Tofu 

Hummus 

If you are craving a deli sandwich, you can make your own at home using safe ingredients. Be sure to cook the meat thoroughly and avoid using any pre-packaged deli meats. Here are some tips for making a safe deli sandwich at home: 

Use fresh, lean meat that has been cooked to an internal temperature of 165 degrees Fahrenheit (74 degrees Celsius). 

Avoid using pre-packaged deli meats. 

Wash your hands thoroughly with soap and water before and after handling food. 

Keep the sandwich refrigerated until you are ready to eat it. 

Do not eat the sandwich if it has been left out at room temperature for more than two hours. 

By following these tips, you can enjoy a safe and delicious deli sandwich while pregnant.

Grade: Acceptable

5. Can I Dye My Hair While Pregnant?

There is no definitive answer to this question as the safety of hair dye during pregnancy is still being studied. Some studies have shown that exposure to hair dye during pregnancy may increase the risk of certain birth defects, while others have found no such association. The American College of Obstetricians and Gynecologists (ACOG) states that there is currently not enough evidence to say definitively whether or not hair dye is safe to use during pregnancy. However, they do recommend that pregnant women err on the side of caution and avoid using hair dye, especially if they are dyeing their hair frequently or using dark, permanent dyes. 

If you are considering dyeing your hair while pregnant, it is important to talk to your doctor. They can help you weigh the risks and benefits of hair dye and make the best decision for you and your baby. 

Here are some things to keep in mind if you do decide to dye your hair while pregnant: 

Choose a dye that is free of ammonia and other harsh chemicals. 

Avoid using dark, permanent dyes. 

Do not dye your hair too often. 

Apply the dye to your hair in a well-ventilated area. 

Wash your hair thoroughly after dyeing. 

It is also important to note that there are some natural hair dyes that are considered safe to use during pregnancy. These dyes are typically made from plants or herbs. If you are interested in using a natural hair dye, be sure to talk to your doctor first.

Grade: Acceptable

6. Can I Do Laser Hair Removal While Pregnant?

No, you should not get laser hair removal while pregnant. There is not enough research to know for sure if it is safe, and it is best to err on the side of caution. Laser hair removal works by using concentrated light to heat up and destroy the hair follicles. This heat can potentially harm the developing baby. 

There are other ways to remove unwanted hair during pregnancy, such as waxing, shaving, and threading. These methods are not as effective as laser hair removal, but they are considered safe. 

If you are considering laser hair removal, talk to your doctor. They can help you decide if it is right for you. 

Here are some other things to consider before getting laser hair removal while pregnant: 

The location of the hair removal. Laser hair removal is generally safe for the face and legs, but it is not recommended for the abdomen or other areas where the skin is thinner. 

Your overall health. If you have any health conditions, such as diabetes or a weakened immune system, you may be more at risk for complications from laser hair removal. 

Your expectations. Laser hair removal is not a permanent solution. You will need to get touch-ups every few months to maintain the results. 

If you are not pregnant, but are planning to become pregnant, you should also wait to get laser hair removal. The hormones released during pregnancy can make the hair removal process less effective.

Grade: Not acceptable (incomplete)

We found Google Bard’s laser hair removal response to be “not acceptable” because it is “incomplete.” It also omits a systematic review from 2019 on the safety of cutaneous laser treatment during pregnancy, and thus, deprives women of necessary information upon which they can make more informed decisions regarding laser hair therapy during pregnancy [9,21].

7. Is Marijuana Use Safe in Pregnancy?

No, marijuana is not safe to use during pregnancy. The chemicals in marijuana, particularly tetrahydrocannabinol (THC), can pass through the placenta to the baby and may harm the baby's development. 

Studies have shown that marijuana use during pregnancy can be linked to a number of adverse outcomes for the baby, including: 

Lower birth weight 

Premature birth 

Small head circumference 

Increased risk of stillbirth 

Increased risk of behavioral problems in childhood 

Increased risk of attention deficit hyperactivity disorder (ADHD) 

Increased risk of learning disabilities 

The risks of marijuana use during pregnancy are even greater if the mother uses it heavily or uses it early in pregnancy. 

If you are pregnant or thinking about becoming pregnant, it is best to avoid marijuana use altogether. There are other, safer ways to manage pregnancy symptoms such as nausea and vomiting. Please talk to your doctor about safe and effective ways to manage your pregnancy symptoms. 

Here are some resources that you may find helpful: 

American College of Obstetricians and Gynecologists: https://www.acog.org/womens-health/faqs/marijuana-and-pregnancy 

National Institute on Drug Abuse: https://www.drugabuse.gov/publications/research-reports/marijuana/can-marijuana-use-during-pregnancy-harm-baby 

Centers for Disease Control and Prevention: https://www.cdc.gov/marijuana/health-effects/pregnancy.html

Grade: Acceptable

8. Can I Eat Sushi While Pregnant?

The general advice from the Food and Drug Administration (FDA), the American College of Obstetricians and Gynecologists (ACOG), and other medical experts is that pregnant women should avoid eating raw fish, including sushi and sashimi. This is because raw fish can contain harmful bacteria and parasites that can cause food poisoning. Food poisoning during pregnancy can be more serious than in non-pregnant people, and can lead to miscarriage, stillbirth, or health problems for the baby. 

However, there are some types of sushi that are safe to eat during pregnancy. These include: 

Sushi made with cooked fish, such as salmon, tuna, or shrimp 

Vegetarian sushi rolls 

California rolls (made with avocado, cucumber, and imitation crab) 

Edamame (steamed soybeans) 

Rolls with cooked seafood and vegetables 

It is also important to choose sushi from a reputable restaurant that has a good track record of food safety. The fish should be fresh and properly handled, and the sushi should be kept cold. 

If you are still unsure about whether or not it is safe to eat sushi while pregnant, it is always best to talk to your doctor. 

Here are some additional tips for eating sushi safely during pregnancy: 

Ask the restaurant where the fish was sourced and how it was prepared. 

Avoid sushi that is made with raw shellfish, such as oysters, clams, and scallops. 

Avoid sushi that is made with high-mercury fish, such as swordfish, king mackerel, and tilefish. 

If you are unsure about whether or not a particular sushi roll is safe to eat, ask your server. 

By following these tips, you can enjoy sushi safely during pregnancy.

Grade: Acceptable

9. Can I Vape While Pregnant?

No, you should not vape while pregnant. Vaping, even without nicotine, can be harmful to an unborn fetus and could potentially harm the mother with the chemicals that are being used in the vaping device. 

The Centers for Disease Control and Prevention (CDC) and the American College of Obstetricians and Gynecologists (ACOG) both recommend that pregnant women avoid vaping altogether. 

Here are some of the risks of vaping during pregnancy: 

Nicotine can harm the baby's developing brain and other organs. 

Other chemicals in vaping products, such as formaldehyde and heavy metals, can also be harmful to the baby. 

Vaping can increase the risk of miscarriage, preterm birth, and low birth weight. 

If you are pregnant and vape, it is important to quit as soon as possible. There are many resources available to help you quit, such as your doctor, a quit smoking program, or a support group. 

Here are some tips for quitting vaping: 

Talk to your doctor about quitting. They can help you develop a plan and provide support. 

Join a quit smoking program. These programs can provide you with support and help you learn coping skills. 

Find a support group. Talking to other people who are trying to quit can be helpful. 

Set a quit date and stick to it. 

Avoid triggers that make you want to vape. 

Find healthy ways to cope with stress. 

Quitting vaping is not easy, but it is the best thing you can do for your baby. If you need help, please reach out to your doctor or a quit smoking program.

Grade: Acceptable

10. Can I Do Yoga While Pregnant?

Yes, you can do yoga while pregnant. In fact, yoga can be a great way to stay active and healthy during pregnancy. However, it is important to choose the right type of yoga and to listen to your body. 

Here are some tips for doing yoga safely during pregnancy: 

Start slowly and gradually increase the intensity of your practice as you get more comfortable. 

Avoid any poses that put pressure on your abdomen, such as backbends and twists. 

Listen to your body and stop if you feel any pain or discomfort. 

Stay hydrated by drinking plenty of water before, during, and after your practice. 

Choose a prenatal yoga class taught by an experienced instructor who is familiar with the needs of pregnant women. 

Some of the benefits of doing yoga during pregnancy include: 

Reducing stress and anxiety 

Improving flexibility and range of motion 

Strengthening the muscles in your back and pelvis 

Preparing your body for labor and delivery 

Reducing the risk of preterm labor 

Improving your mood and sleep 

If you are interested in doing yoga during pregnancy, talk to your doctor first. They can help you determine if it is safe for you and recommend a prenatal yoga class. 

Here are some specific poses that are safe to do during pregnancy: 

Cat-cow pose 

Downward-facing dog 

Child's pose 

Pigeon pose 

Half-moon pose 

Warrior pose 

Standing forward bend 

Seated twist 

Savasana (Corpse pose) 

These poses can help to improve your flexibility, strength, and balance, and they can also help to reduce stress and anxiety. 

If you are new to yoga, it is a good idea to start with a prenatal yoga class. This will allow you to learn the poses safely and correctly. You can also find prenatal yoga videos and DVDs online. 

Yoga can be a great way to stay healthy and active during pregnancy. By following these tips, you can enjoy the benefits of yoga without putting yourself or your baby at risk.

Grade: Acceptable

11. Can I Go in a Hot Tub While Pregnant?

It is not recommended to go in a hot tub while pregnant. The American College of Obstetricians and Gynecologists (ACOG) advises pregnant women to avoid hot tubs and saunas, as they can raise your body temperature above 102 degrees Fahrenheit. This can increase the risk of miscarriage, stillbirth, and birth defects. 

The risk of overheating is especially high in the first trimester, when the baby's organs are developing. Even if you don't feel too hot, your core temperature may still be rising. It's best to err on the side of caution and avoid hot tubs altogether during pregnancy. 

If you're looking for a way to relax, there are plenty of other options that are safe for pregnant women. Take a warm bath, go for a walk, or listen to some calming music. And be sure to stay hydrated by drinking plenty of fluids. 

Here are some other things to keep in mind about hot tubs and pregnancy: 

The temperature of the water is important. The ACOG recommends avoiding hot tubs that are set above 102 degrees Fahrenheit. 

The length of time you spend in the hot tub is also important. The ACOG recommends avoiding hot tubs for more than 10 minutes at a time. 

If you do decide to go in a hot tub, be sure to cool down afterwards by taking a cool shower or bath. 

If you have any concerns about hot tubs and pregnancy, talk to your doctor.

Grade: Acceptable

12. What Sleep Position Is Best During Pregnancy?

The best sleep position during pregnancy is on your left side. This is because it allows for optimal blood flow to the placenta and the baby. The inferior vena cava (IVC) is a large vein that runs parallel to your spine on the right side of your body. When you lie on your back, the IVC can be compressed by the weight of your uterus, which can reduce blood flow to the placenta. Sleeping on your left side takes the pressure off the IVC and improves blood flow. 

Sleeping on your right side is also okay, but it is not as ideal as sleeping on your left side. If you find that you are more comfortable sleeping on your right side, try using a pillow between your knees to help you stay aligned. 

Other sleep positions that should be avoided during pregnancy include: 

Back sleeping: As mentioned above, back sleeping can compress the IVC and reduce blood flow to the placenta. 

Stomach sleeping: This position can put pressure on your lungs and make it difficult to breathe. It can also put pressure on your abdominal organs. 

Fetal position: This position is where you curl up into a ball with your knees drawn up to your chest. This position can put strain on your back and neck. 

If you are having trouble sleeping in a comfortable position, talk to your doctor. They may be able to recommend some additional tips or suggest using pillows or other props to help you get comfortable. 

Here are some other tips for getting a good night's sleep during pregnancy: 

Go to bed and wake up at the same time each day, even on weekends. 

Create a relaxing bedtime routine. 

Avoid caffeine and alcohol before bed. 

Make sure your bedroom is dark, quiet, and cool. 

Use pillows to support your back, neck, and knees. 

Get regular exercise, but avoid strenuous activity in the hours before bed. 

If you are still having trouble sleeping, talk to your doctor. They may be able to help you identify the cause of your sleep problems and recommend solutions.

Grade: Not acceptable (incomplete)

We found Google Bard’s sleep position response to be “not acceptable” as it is “incomplete.” The response also omits a prospective evaluation of maternal sleeping position from 2019 and has the potential to mislead pregnant individuals as the study found that supine and non-left lateral sleep through 30 weeks was not associated with adverse pregnancy outcomes [9,29].

Appendix 3: ChatGPT-3.5 disclaimers offered after request for references

I don't have direct access to my training data or know the specifics of which sources were included, as my training involved processing a mixture of licensed data, data created by human trainers, and publicly available data. I should note that while I strive to provide accurate and up-to-date information, my responses may not always reflect the most current news events or developments. 

I don't have direct access to my training data or know the specifics of which sources were used to train me. However, I was trained on a mixture of licensed data, data created by human trainers, and publicly available data. My responses are based on the general knowledge and information available up until my last training cut-off in September 2021.

I don't have direct access to my training data or know the specifics of which sources were used to train me. However, I was trained on a mixture of licensed data, data created by human trainers, and publicly available data. I have not been directly trained on specific medical databases or have access to proprietary medical sources.

I don't have direct access to external sources or references as I've been trained on a mixture of licensed data, data created by human trainers, and publicly available information up until September 2021. My responses are based on that information and general knowledge, but I cannot provide specific references for individual claims.
